# Improving wild animal welfare through contraception

**DOI:** 10.1093/biosci/biae071

**Published:** 2024-09-11

**Authors:** Simon Eckerström Liedholm, Luke Hecht, Vittoria Elliott

**Affiliations:** Wild Animal Initiative, Minneapolis, Minnesota, United States; Department of Biosciences, Durham University, Durham, England, United Kingdom; Wild Animal Initiative, Minneapolis, Minnesota, United States; Wild Animal Initiative, Minneapolis, Minnesota, United States; National Museum of Natural History, Smithsonian Institution, Washington, DC, United States

**Keywords:** fertility control, density dependence, population dynamics, population management, ecological feedback

## Abstract

To date, research on the welfare impacts of wildlife contraceptives has mostly been focused on the potential harms of contraceptives. However, there are compelling theoretical reasons to expect direct and indirect welfare benefits of wildlife contraceptives. These positive welfare effects would be experienced by more than just the treated individuals, because per capita resource availability will increase with decreasing numbers of individuals sharing a resource. In the present article, we discuss the potential for wildlife contraceptives to alleviate resource competition and their associated negative welfare effects at different scales. These effects are expected to vary across contexts and would presumably be stronger when wildlife contraceptives are used with the explicit purpose of improving wild animal welfare. The potential for considerable welfare gains for wildlife through the targeted use of contraceptives highlights the importance of both species-specific studies on the welfare benefits of wildlife contraceptives and further research on the links between population dynamics and wild animal welfare.

Wildlife contraception is an accepted method for controlling populations of many wild animal species (Kirkpatrick et al. 2011). Although the use of wildlife contraceptives may improve wild animal welfare (see box 1 for our definition of *welfare*), research related to the welfare impacts of wildlife contraceptives has to date largely been focused on understanding and mitigating harmful side effects (Gray and Cameron 2010), as opposed to identifying and harnessing possible welfare benefits. Although these studies are laudable and indispensable, they have not been specifically targeted at understanding the potential welfare benefits of wildlife contraceptives. Crucially, however, there are compelling theoretical arguments that suggest that there could be welfare benefits of administering wildlife contraception in certain contexts because of ecological feedback effects (Ransom et al. 2014). These effects are anticipated to be both direct, by reducing the energetic investment into reproduction (Stearns 1989), and indirect, by generally reducing population densities and by reducing sibling competition through reductions in the number of offspring, therefore lowering resource competition (Andersen et al. 2011, Prevedello et al. 2013, Ruffino et al. 2014). Therefore, welfare benefits could be experienced beyond the treated individuals, because contraception might increase the per capita resource availability by decreasing the number of individuals sharing a resource (Sinclair and Krebs 2002, Prevedello et al. 2013). Although this suggests that wild animal welfare benefits could occur in many contexts, few studies have looked for direct evidence of positive welfare effects on wild animals (Soryl et al. 2021). This problem is exacerbated by the fact that assessing to what extent different proxies of welfare (e.g., body condition or disease symptoms) ultimately reflect the actual valence of affective states (see box 1) can be hard and labor intensive (Browning 2022). In the present article, we describe the theoretical principles behind when and how we might expect wildlife contraception to positively affect wild animal welfare. We explore relationships among demography, life history trade-offs, density dependence, and wild animal welfare and make the case for more theoretical and applied research on the welfare effects of wildlife contraception. By exploring how decreasing population densities through wildlife contraceptives could increase per capita resource availability, we also aim to contribute to the understanding of complex wildlife dynamics.

## Density-dependent welfare

Because vital resources such as food, water, and shelter are limited in any natural system, a population of animals cannot grow indefinitely and will eventually be limited by resource availability (Sibly and Hone 2002) if it is not first arrested by top-down effects, such as predation. The collective effect of these limits to population growth is usually described as *density dependence* (box 1). Evidence for density-dependent limits to population growth come from both theory (Murray 1994, Sibly and Hone 2002, Lebreton and Gimenez 2013, see Tanner 1966 for a review of earlier studies) and empirical studies (red squirrels, Wauters and Lens 1995; elk, Sauer and Boyce 1983; wildebeests, Owen-Smith 2006; ungulates, Gaillard et al. 2000; voles, Turchin and Ostfeld 1997, Henttonen and Hanski 2000; grasshoppers, Belovsky and Joern 1995; see Sinclair 1996 for a summary). In particular, high population densities have been shown to correlate with lower body condition (Mugabo et al. 2013) and lower survival (Sibly and Hone 2002, Cowan and Massei 2008), which, in turn, are likely to affect welfare negatively. High densities have also been observed to lead to high physiological stress in voles (Shang et al. 2022), which can result in poor welfare. In some contexts, welfare-associated impacts have been reported in relation to density-dependent effects of disease. For example, chronic wasting disease in deer (Habib et al. 2011), white-nose syndrome in bats (Langwig et al. 2012), and tuberculosis in wild boars (Tanner et al. 2019) have been shown to exhibit density-dependent

Box 1. **Glossary**

**Welfare**. We define welfare as the valence of the affective state (Posner et al. 2005) of a sentient animal (i.e., experiences as perceived by the individual; Duncan 2004). In other words, our definition of *welfare* is essentially equivalent to the fifth domain of the five domains model, the mental domain (Mellor et al. 2009). It should be noted that the health, nutritional state, abiotic and biotic environmental conditions, and behavior combine to determine an animal's welfare. In other words, they are all important predictors and contributors to the valenced affective states (i.e., welfare) of an animal.

**Carrying capacity**. We define *carrying capacity* as the population size that is reached when all negative density-dependent effects are accounted for, where such density-dependent effects would cause the population to exhibit net-zero growth (Sibly and Hone 2002), assuming no migration. Because of fluctuations, the population size may overshoot or undershoot the carrying capacity, such that the population size does not exactly equal the carrying capacity for an extended period of time (i.e., the carrying capacity acts as an attractor state).

**Population density**. *Population density* refers to the ratio between the observed size of a population and its carrying capacity—that is, the maximum number of individuals that can be sustained.

**Density-dependent welfare**. If welfare is density dependent, then the average welfare in a population is expected to change as population density changes. It is important to note that density-dependent welfare effects can occur simply through a change in resource levels, for instance, despite the number of animals in the population staying the same.

effects. Many populations of wild animals appear to exist close to their carrying capacity (Sibly et al. 2005) and are frequently affected by resource scarcity (Prevedello et al. 2013, Ruffino et al. 2014). Resource scarcity likely affects the average welfare of wild animals directly—for example, through effects on body condition (Meagher 2009, Grandin 2021) from starvation and dehydration during droughts (Gregory 2004). It likely also has indirect effects on welfare as a result of ensuing physical weakness (Gordon et al. 1988), which can make it harder to find appropriate shelter, potentially increasing the risk of exposure (Sibly and Hone 2002, Hill et al. 2019). Although the prevalence of starvation may seem low solely on the basis of reports of direct causes of death (Hill et al. 2019), starvation also increases the risk of other causes of death (Bartmann et al. 1992), so it is reasonable to predict that starvation as a source or contributor to mortality is fairly widespread. Furthermore, starvation is associated with a negatively valenced affective state (i.e., experiencing hunger and thirst is unpleasant; Gregory 2004). By this rationale, alleviating resource scarcity could therefore directly (e.g., through reduced starvation risk) and indirectly (e.g., through reduced disease susceptibility) improve the welfare of wild animals. Resource scarcity could also be *temporarily* alleviated by artificially increasing the total amount of resources; however, this would lead to higher survival and birth rates, which, in turn, would lead to compensatory population growth, leaving competition for resources and welfare effects largely unchanged (Prevedello et al. 2013, Ruffino et al. 2014). Targeting the reproductive rate instead of or in addition to food supplementation constitutes a more sustainable way to alleviate resource scarcity.

**Figure 1.**
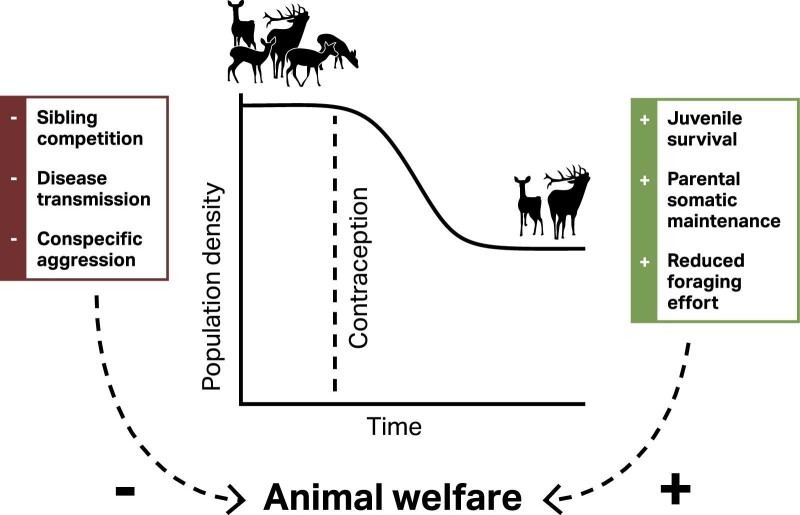
Potential benefits to wild animal welfare from a reduction in population density through the use of wildlife contraceptives.

Artificially reducing the birth rate has been shown to increase survival (Kirkpatrick and Turner 2007, Williams et al. 2007), suggesting that welfare might be positively affected as well (Ellis et al. 2012). Juveniles seem to particularly benefit from reduced competition for resources (Davis and Pech 2002), so welfare improvements from wildlife contraception are especially likely in species where early life stages are characterized by low survival (Sol et al. 1998, Halley et al. 2018, Healy et al. 2019, Hecht 2021) and where juvenile survival is density dependent (Arcese et al. 1992, Gaillard et al. 1998, Armstrong et al. 2005, Bailey et al. 2010, Payo-Payo et al. 2016). There is also some evidence of wildlife contraceptives improving body condition—for example, in horses (Turner and Kirkpatrick 2002, Kirkpatrick and Turner 2007), Soay sheep (Tavecchia et al. 2005), rabbits (Williams et al. 2007), and white-tailed deer (McShea et al. 1997, Gionfriddo et al. 2011), as well as increased survival (see e.g., Ramsey 2005). If a reduction in the per capita reproductive rate leads to a decrease in resource competition and an increase in adult and juvenile survival, the average welfare of a population will likely increase (figure 1).

Culling, hunting, poisoning, and trapping (i.e., methods that increase the mortality rate instead) have also been used to artificially reduce population densities. It is therefore likely that the aforementioned welfare benefits of reduced densities associated with wildlife contraceptives, will apply to these methods as well. However, methods that increase the mortality rate specifically of adults can be associated with a downward shift from adult to juvenile life stages, which might have negative effects on overall welfare (Hecht 2021). The immediacy of death and the degree of potential welfare impacts of such methods differ depending on the methods used and the species targeted (Littin et al. 2014, Hampton et al. 2015, Allen et al. 2019), as well as what the counterfactual fate of the individual would have been. It should be noted that even if they may provide welfare benefits for the remaining individuals, culling and related methods are increasingly seen as unethical when alternatives exist to achieve the same outcomes (Barfield et al. 2006, Degeling et al. 2016, Feber et al. 2017).

## Context-dependent effects

There may be circumstances that are worse or better suited to achieving welfare benefits of artificially reducing population densities through wildlife contraction (table 1).

**Table 1.** Examples of circumstances in which contraception is likely beneficial versus neutral or detrimental to welfare.

**Attribute**	**Contraception possibly beneficial to welfare**	**Contraception possibly not beneficial to welfare**
Sociality	Solitary	Cooperative group living
Population density determinants	Bottom up (e.g., food)	Top down (e.g., predation)
Reproductive constraints	Weak	Strong (e.g., stronger density dependence in reproduction than survival)
Counterfactual energy investment	Somatic maintenance or survival	Extended mating behavior

### Population dynamics and life history

Populations that exist near their carrying capacity and are regulated by resource availability are likely to benefit more from wildlife contraception than are populations regulated in other ways, such as by nest-site availability (Pöysä and Pöysä 2002), abiotic factors (e.g., recurring severe weather; Hamel et al. 2010), or predation (Tanner 1966), particularly when these factors are unrelated to population density. Welfare benefits are also less likely where individuals naturally reduce reproduction at high densities (Barlow et al. 1997) through, for example, skipping reproduction (Hamel et al. 2010, Rideout and Tomkiewicz 2011), or when the environmental conditions are generally unsuitable for ensuring parental or offspring survival. Finally, welfare benefits are potentially less likely to materialize when energy freed up from reproduction by contraception, instead of being invested in welfare-enhancing traits such as somatic maintenance, is invested in other traits that may decrease welfare or that otherwise have unclear effects on welfare, such as mating displays and competition for mates (Ji et al. 2000), or secondary sex characteristics (McShea et al. 1997, Ji et al. 2000, Fraker et al. 2002). Such traits are generally energetically costly and consequently reduce the resources available for somatic maintenance that is commensurate with increased welfare.

The average welfare benefits may also depend on changes in the demography of the population—if the average welfare differs between juveniles and adults, for instance (Hecht 2021). Would-be parents with the largest contrafactual reduction in offspring numbers are likely to experience the greatest relaxation of resource constraints and, therefore, a possible increase in welfare from contraception. Artificially limiting the number of offspring produced leads to an overall reduction in parental energetic investment and preserves more resources for their own somatic maintenance (Kirkwood 1977, Stearns 1989, Kirkwood and Rose 1991, Lemaître et al. 2015). Therefore, the sex with the largest parental investment is also likely to experience the largest welfare benefit from contraceptives (cf. Robbins 1993). Similarly, because of reduced sibling competition, there will be more resources available per individual as long as some offspring are born (Mendl 1988, Andersen et al. 2011, Hudson et al. 2011). In general, many life-history traits are likely to differentially affect the potential welfare benefits of wildlife contraception, such as the degree of parental care, maturation age, fecundity, and dispersal patterns; future studies should explore the conditions under which these welfare effects are modulated by life-history traits.

### Social systems

Some social species living in groups could be negatively affected by artificial population reductions, if smaller groups perform worse (Angulo et al. 2017, Lerch et al. 2018)—for example, where tasks such as guarding and hunting require a minimum group size to be effective because of the need for the division of labor or to share social information (Angulo et al. 2017). For instance, an average pack of African wild dogs (*Lycaon pictus*) or meerkats (*Suricata suricatta*) would potentially be worse off if the group size was reduced (Clutton-Brock et al. 1999, Courchamp and MacDonald 2001). It might be expected, therefore, that the welfare benefits of artificially reducing fertility could be large when fierce competition among individuals for resources is prevalent but small or even negative when cooperative behavior is beneficial for resource exploitation and individual survival. In some group-living species, particularly highly social apex predators, dominant individuals often control the reproduction of conspecifics, such that population densities stay well below the point where resources become limiting (Wallach et al. 2015). Contraception in these cases might therefore be less likely to be beneficial to their welfare.

## Future directions

Before making management recommendations, it is essential to better understand the potential differences in welfare effects of wildlife contraceptives for individuals of different species and populations, whether and how life-history strategies modulate such effects, the relationship between welfare and resource availability, and the potential for negative or positive indirect effects on the welfare of nontarget species. Future research aimed at identifying where wildlife contraception can be most beneficial will therefore need to consider all of these factors but especially how wild animal welfare is related to density-dependent effects, as well as how to avoid harmful side effects of wildlife contraceptives (Nettles 1997, Gray and Cameron 2010). Finally, researchers could also explore these questions in species not yet treated with wildlife contraceptives, if benefits to wild animal welfare seem plausible and additional economic costs are small relative to business as usual.

## Conclusions

Reducing population densities through the use of wildlife contraceptives can trigger changes in traits such as survival and body condition, potentially leading to welfare benefits for animals in the treated population. Such benefits can be accumulated both at the within-family level by reducing sibling competition and decreasing the cost of reproduction for parents and at the population level by reducing intraspecific competition among unrelated individuals. In the present article, we have provided an overview of key traits and contexts under which wildlife contraception is most likely to provide welfare benefits, as well as those likely to be associated with neutral or negative effects on welfare. Future studies that consider potential positive effects of wildlife contraception for the welfare of wild animals will likely also contribute to our understanding of how resource scarcity and density dependence interacts with life history, demographic patterns, and other population dynamic processes. In so doing, these studies will help build valuable new connections between population ecology on one hand and animal welfare science and population management on the other. We encourage both applied and theoretical researchers to test these hypotheses and further clarify under what circumstances wildlife contraception might provide welfare benefits to wild animals. Greater knowledge of the individual and population-level effects of wildlife contraceptives will eventually present us with the opportunity to meaningfully improve wild animal welfare.

## Author Biography

Simon Eckerström Liedholm (simon.liedholm@gmail.com), Luke Hecht, and Vittoria Elliott are affiliated with the Wild Animal Initiative, in Minneapolis, Minnesota, in the United States. Luke Hecht is also affiliated with the Department of Biosciences at Durham University, in Durham, England, in the United Kingdom. Vittoria Elliott is also affiliated with the National Museum of Natural History, at the Smithsonian Institution, in Washington, DC, in the United States.
